# Effects of Lead on IQ in Children

**DOI:** 10.1289/ehp.114-a85

**Published:** 2006-02

**Authors:** Claire B. Ernhart

**Affiliations:** Case Western Reserve University, Strongsville, Ohio, E-mail: cxe@po.cwru.edu

Lanphear et al. (2005) pooled data from seven prospective studies that had been initiated to test the effect of prenatal and early childhood lead exposure. The primary investigators of these studies had planned the studies so that the sequence of data collection might shed light on the question of early causation. At that time, most of us anticipated a strong association of prenatal exposure and developmental deficit related to rapid prenatal central nervous system (CNS) development (Ernhart 1992). Lanphear et al. (2005) pooled our data to report a significant association of cord blood lead (BPb) and IQ (intelligence quotient) and concluded that prevention of lead exposure must occur before pregnancy or childbirth. Their analysis did not include control of the sociodemographic factors known to confound research on the topic; hence, the conclusion is not justified.

In the balance of the report, Lanphear et al. (2005) selected concurrent lead level at 5–6 years of age, as opposed to earlier measures of lead exposure, because it had the highest association with IQ. The closer association for the lead measurement made at or near the time of the IQ test may reflect concomitant factors not well controlled in the analyses. In most studies, parental intelligence and HOME (Home Observation for Measurement of the Environment; a measure of caretaking and parental stimulation) are major predictors of child IQ. These variables are difficult to measure (Kaufman 2001), and undercontrol of confounding is likely. Bias is particularly likely in the data of the Rochester, New York, cohort (Canfield et al. 2003) because the HOME (toddler version) was administered at the age of 2 years, not at 5–6 years of age.

Using available covariate data, Lanphear et al. (2005) did report a deficit of approximately 2 IQ points for the BPb range of 10–20 μg/dL. This replicates previous analyses conducted by Pocock et al. (1994). The latter investigators interpreted the association as possibly due to limited control of confounding, selection biases, and/or reverse causality.

The most problematic portion of the article by Lanphear et al. (2005) concerns very low lead exposure. The authors selected data for the 244 children who had peak, or maximal, BPb levels < 10 μg/dL. The decline in IQ for this group consisted of 6.2 points for the concurrent BPb range of 1–10 μg/dL (β = −0.80, SE = 0.48, *p* = 0.09). For a more restricted group of 103 children with peak BPb levels < 7.5 μg/dL, the association was stronger (β = −2.94, SE = 1.14, *p* = 0.012) although the sample size was further truncated. Lanphear et al. (2005) concluded that “lead exposure in children who have maximal BPb levels < 7.5 μg/dL is associated with intellectual deficits.” There are major problems with this conclusion.

First, groups selected on the basis of peak lead level < 10 μg/dL and < 7.5 μg/dL differed significantly from the balance of the sample on factors omitted as non-contributing for the full study. Lanphear et al. (2005) ignored race (U.S. cohorts), maternal age, and maternal use of cigarettes and alcohol during pregnancy in the analyses of these groups.

Second, cohort contribution was critical for these groups. Of the 103 children with BPb levels < 7.5 μg/dL (Lanphear et al 2005), 67% were from the Rochester cohort. In addition to the limitation in the HOME data, information regarding this cohort at 3 and 5 years of age reflects peculiar shifts in demographic variables, including race and maternal education (Canfield et al. 2003; Canfield RL, Henderson CR, Lanphear BP, Cory-Schlecta DA, Smith EG, Cox C, unpublished data). This was a prospective study, yet the sample increased from 154 children at 5 years of age to 182 at 6 years of age, and the number with peak lead levels < 10 μg/dL increased from 86 to 103. Canfield et al.’s 6-year data used in the pooled analysis have not been published, and my requests for further information were denied.

Finally, there was no significant association of IQ and three of the four indices of lead exposure—early childhood, peak (or maximal), and lifetime average—for the segments of the sample with peak lead levels < 10 μg/dL or < 7.5 μg/dL. Lanphear et al. (2005) omitted these analyses from their article.

Lanphear et al. (2005) reached conclusions intended to support policies to further reduce the already low level of childhood lead exposure. Although I contributed data [the Cleveland Study (Ernhart et al. 1989)] and participated in planning and review of analyses, I withdrew from authorship because I could not concur with the manuscript, including the inferences drawn.
